# Syrup of Iodide Potassium in Syphilis

**Published:** 1860-01

**Authors:** 


*Syrup of Iodide of Potassium in Syphilis.*

M. Basin gives iodide of potassium in this disease, in doses of five to seven and a half grains, till seventy-seven grains are given. Seldom had occasion to give more. He prefers the following formula:—

℞ Bi iodide of Mercury, 3 grains.
Iodide of Potassium, 2½ drachms.
Syrup of Saponaria, 18 ounces.

Dose—Begin with two teaspoonsful, twice a day, and increase until four teaspoonsful are taken at a time.—*Journal of Materia Medica.*

